# Half-Yearly Report of Cases in Midwifery, Which Have Occurred in the Northern District of the London and Southwark Midwifery Institution

**Published:** 1830-08

**Authors:** C. Waller

**Affiliations:** Consulting Accoucheur to the above Institution, and Lecturer on Midwifery at the Medical School, 58, Aldersgate street.


### THE LONDON
### Medical and Physical Journal.

No. 378, VOL. LXIV.] AUGUST 1830. [No. 50, *New Series.*

For many fortunate discoveries in medicine, and for the detection of numerous errors, the world is indebted to the rapid circulation of Monthly Journals; and there never existed any work to which the Faculty, in Europe and America, were under deeper obligations than to the *Medical and Physical Journal of London*, now forming a long but an invaluable series.—*Rush.*

### ORIGINAL PAPERS, AND CASES,
### OBTAINED FROM PUBLIC INSTITUTIONS AND OTHER AUTHENTIC SOURCES.

### MIDWIFERY.

*Half-yearly Report of Cases in Midwifery, which have occurred in the Northern District of the London and Southwark Midwifery Institution.* By C. WALLER, Esq. Consulting Accoucheur to the above Institution, and Lecturer on Midwifery at the Medical School, 58, Aldersgate street.

| 1830. | No. Women delivered. | Sex of Children: Males. | Females. | Born alive. | Stillborn. | Presentation. |
|---|---|---|---|---|---|---|
| January .. | 29 | 14 | 15 | 28 | 1 | 27 Natural<br>1 Breech<br>1 Face to Pu. |
| February .. | 23 | 14 | 9 | 22 | 1 | 22 Natural<br>1 Breech |
| March .... | 35 | 21 | 14 | 33 | 2 | Natural |
| April .... | 25 | 9 | 16 | 23 | 2 | 24 Natural<br>1 Arm |
| May...... | 43 | 24 | 19 | 39 | 4 | 39 Natural<br>1 Foot, 2 Br.<br>1 Face |
| June..... | 26 | 14 | 12 | 24 | 2 | 25 Natural<br>1 Foot |
| Total .... | 181 | 96 | 85 | 169 | 11 | |

*** Several of the above, reported as Stillborn, were Premature Births.

IN the Report of the preceding half-year, I mentioned that several cases of abdominal inflammation had occurred, but which were of the active character; and accompanied with considerable vascular power, and therefore easily subdued by a corresponding activity of treatment: I am sorry the report this time must be much more unfavorable; puerperal fever, in its most malignant form, having, in the early part of the year, attacked several of our patients, and in many instances, in spite of (I trust) the best-directed efforts, terminated in death.

It is much to be regretted that no efficient remedy has been discovered for this horribly devastating malady, and that we do not even yet agree as to its precise nature. That it is of an inflammatory character, is evident enough from the post-mortem appearances; but what is the specific nature of this inflammation, is a question which has yet to be settled. It is, I conceive, sufficiently obvious that it is *not* acute inflammation of the peritoneum: it differs from it in its characters during life, and in its appearances after death; the chief difference in the living subject consisting in the sudden and extraordinary rapidity of the pulse, marked also by a very peculiar undulating beat, widely differing from the full and round, or contracted and hard throb which occurs in peritonitis. It is not uncommon to find the pulse mounting up to 130 in the minute, within an hour or two after the attack; and, in the late epidemic, acute pain in the abdomen was, with one or two exceptions, by no means the most prominent symptom. There is also more cerebral affection, and, in many instances, an immediate and irremediable prostration of the powers of the constitution.

Then, with regard to the dissection of these cases, we find, it is true, a copious effusion; but it is of a muddy appearance, mixed with a large quantity of substances resembling curd; and where this substance is deposited between the folds of the intestines, there must be, of necessity, a certain degree of agglutination, but I have never seen those tight adhesive bands which are so commonly observable after acute abdominal inflammation.

The inflammation appears to be migratory in its progress. Several well-marked cases have occurred in which the uterus was evidently the part first affected; after which, it extended itself upwards till it reached the diaphragm, the patient eventually sinking from the same kind of disease attacking the pleura, producing the same kind of effusion within its cavity. Here, also, those firm adhesive bridles which are so frequently seen uniting the two pleural surfaces were wanting, though the curdy matter was deposited in large masses. The mode in which the two diseases terminate when they end in recovery, is also different: in acute inflammation, we can frequently knock down the disease at once, the remedies almost immediately putting a stop to the pain, and equalizing the balance of the circulation; whilst, in puerperal fever, it continues for several days, gradually, and not rapidly, disappearing; the plan of treatment merely keeping it within bounds, as it were, and thus allowing the system slowly to recover itself.

Another interesting question arises, whether it be of an infectious nature? and, if so, by what means the contagion is conveyed from the one to the other. The information I have obtained from my own experience, and from that of others, appears to turn the scale in favor of the opinions of those who believe that the disease may be carried from one to another; but I strongly suspect that contact with the genitals is the method by which it is conveyed. A very strong argument in favor of this opinion is the fact that, although I visited seven or eight times in the day various puerperal patients, who were labouring under, and in fact dying from, this fever, in no single instance was it communicated to my private patients. It should be remembered that in very few instances was the *delivery* conducted by myself, and therefore there was no necessity for my making any vaginal examinations; and, in most of the dissections after death, I avoided, as much as possible, coming into actual contact with the diseased parts.

My own opinions regarding the nature of the disease are scarcely sufficiently mature to justify me in making them public, and it is rather with a view of eliciting information for myself than of conveying it to others, that I wish to call the attention of the profession to the subject.

The London and Southwark Midwifery Institution has not been the only one that has suffered from the ravages of puerperal fever; nor has it been confined to public charities: very many accoucheurs must have lately had opportunities of seeing it; and I cannot help thinking that, if the symptoms, progress, and treatment of the disease, together with the appearances after death, where this event has taken place, were faithfully recorded, that something like a knowledge of its true nature might be procured: then, and not till then, can we expect to find out a successful method of treatment.

In private practice I have seen two cases only: the first was the wife of a surgeon, living at the West end of the town, whom I was requested to visit in consultation on the fifth day of the disease. She had at the onset been very actively treated, and was then in a state of alarming collapse, complaining still of great pain, with considerable tension of the abdomen; the discharges were very offensive, and there was no secretion of milk; the head was much affected; the stomach very irritable, and the bowels rather constipated; the pulse rapid. The plan of treatment consisted in attending to the bowels, in soothing the stomach, in employing counter-irritation to the abdomen, by means of a bag containing chamomile flowers, the outside of which was wetted with oil of turpentine, and afterwards in instituting a gentle mercurial course. The oil of turpentine was used internally as a purgative with considerable advantage, given in doses of six drachms, floated upon cinnamon water. This patient was in a precarious state for some weeks, and was reduced to a mere skeleton; but eventually regained her health, strength, and plumpness. The second case terminated fatally: it was that of a young female, who was delivered after a tolerably easy labour. About an hour previous to its completion, she complained of great chilliness, and the pulse flagged a little. Pulv. Secalis ℈j. was given, and a little warm brandy and water. The coldness was not, however, removed, but remained till she was placed in bed, about an hour after the birth of the child. On the following day she seemed doing well, although she complained of more after-pains than usual. On the third morning I found her very ill: she complained greatly of pain in the head, the countenance was flushed, the scalp hot, and the pulse was 140 in the minute. Ordered her twelve leeches to the temples, and an aperient. In the evening the head was relieved, but there was considerable abdominal uneasiness when pressure was applied. She remained much in the same state, as far as the pulse was concerned; sometimes referring the pain to the side of the abdomen, at others to the chest; her tongue was not, at first, much altered, but it soon became very dry and glossy, and the bowels usually relaxed. Mercury, so as to affect the mouth, was given, but produced no amelioration of symptoms. On the seventh day after delivery, I was summoned in great haste, and found her with a very rapid pulse, gasping for breath, and suffering much from palpitation of the heart. As there seemed to be a good deal of hysterical excitement, I ordered her Sp. Amm. fœtid. cum Mist. Camph.; and this was productive of so much temporary benefit, that she begged it might be repeated the next day. In the course of the disease, digitalis was administered and blisters applied; but she died on the eleventh day. On examining the body, the intestines were seen to be inflated, and there was a slight effusion of limpid serum into the abdominal cavity, but no other marks of inflammatory action. There was most extensive disease in the chest: the pleura covering the lungs was greatly thickened, so as to form a very dense membrane, evidently the result of disease of long standing. The costal pleura was injected with blood, which was dark coloured, appearing in patches like small ecchymoses. There was a very large effusion of turbid serum, in which flakes of curd-like substance were mixed in abundance; there was also a considerable quantity of this substance deposited upon the surface of the pleura, but not adherent to it. I find, on reference to my Adversaria, that it is described as "exactly resembling that which takes place in the abdominal cavity in the worst forms of puerperal fever;" and I have no doubt that it was an inflammation of precisely the same nature, varying only in its seat; the pleura, instead of the peritoneum, being the part attacked. These two cases occurred before those of the Institution, about to be related.

Case I. Mrs. B. æt. thirty-five, was delivered of her fourth child, after a very easy labour, on the 20th of January. During the night she was teazed with efforts to vomit, though but little was ejected. There were no after-pains, the pulse small and soft. On the 21st, these efforts continued: she had taken an egg to her breakfast, which perhaps tended to increase them. A little weak brandy and water was given her, and small doses of Tinct. Opii.

22d.—More easy and tranquil: the retching returns by fits; the bowels rather confined. Capiat Ol. Ricini ℥ss. cras mane.

23d.—In the night purging came on: she nevertheless took the oil. She complained of tenderness over the uterine region; the pulse 120. Hot cloths were applied, and calomel and opium administered.

24th.—Pain in the belly has subsided; vomiting returned; the tongue is white, and thickly coated; there is considerable difficulty of breathing, with some pain in the chest. A blister was ordered, and the saline draught in a state of effervescence. In the evening, she was evidently sinking; dyspnœa increased; the pulse at the wrist not to be felt; the caroid pulsations were 140 in the minute; there was rattling in the throat, which continued till one o'clock on the following morning, when she expired.

*Sectio cadaveris, thirty-six hours after death.* A small quantity of transparent serum was observed in the cavity of the abdomen; there was no turgescence of the vessels of the peritoneum; the round ligaments were highly injected, and one of the ovaries converted into a substance exactly resembling, in its external character, very pure and white hog's lard, in which there was also a quantity of hair. The spleen was very soft, almost rotten in its texture, the slightest force breaking it down. In the thoracic cavity was a copious turbid effusion, mixed with that curdy substance so commonly witnessed after puerperal inflammation. The vessels of the bronchial membrane were very turgid.

This patient was of a remarkably relaxed and feeble constitution; her countenance was naturally pale, almost to ghastliness; and she had been for some time troubled with dyspnœa. She was the first patient upon whom I performed transfusion, (about five years since:) no trace of the operation could be discerned, though the vein was carefully examined.

Case II. Mrs. B., æt. twenty-five, was delivered of a stillborn child on the 28th of January. The fœtus appeared to have been dead for some time, for it was quite putrid. Immediately after the delivery, a quantity of clotted blood was discharged. Capiat Mist. anodyn.

29th.—Appears to be doing well. Not having had much sleep, the mixture was ordered to be repeated.

30th, six p.m.—Last night she had a rigor, followed by heat and profuse perspiration; afterwards there was pain in the uterine region, which is now very tender to the touch; there was also a soreness complained of in the bowels, which was not aggravated on pressure; pulse 120, and by no means strong; tongue clean; no milk. Applic. abdom. Hirudines xx., et sumat quartis horis Hydr. Submur. gr.ij.; Pulv. Opii gr. iss.

Eleven p.m.—Much the same. Some of the leeches not yet come off, and no pill had been taken.

31st, eight a.m.—Free from pain; pulse 120, and soft; head much confused; is very drowsy, but easily roused. Answers questions rather incoherently, (partly, probably, the effect of the opium.) Bowels opened three times during the night.

Noon.—Still drowsy and confused. Complains of slight pain when pressure is made on the uterus; tongue brown; sordes about the mouth and gums; urine scanty and high coloured; no lochial secretion.—At eight o'clock, not having had a motion, she took Ol. Terebinth. ℥ss.

Ten P.M.—The turpentine has operated once. She is in no pain, but is much confused in her head.

February 1st.—Had four copious watery evacuations during the night. Head not so much confused; pulse 110; no pain in the abdomen. Capiat Sodæ Tart. ʒi.; Syr. Papav. ʒss.; Aq. Menthæ pip. ʒxi. quartis horis.

In the evening, she complained of pain at the back of the chest; the respiration was difficult, and she experienced that peculiar sensation in the throat called *globus hystericus*. Capiat Hyd. Submur. gr. iij.; Opii gr. i. horâ somni.

2d, mane.—Has passed a very restless night. Bowels much relaxed, the motions black and offensive; abdomen tense, and tender on pressure; respiration quicker, but not so difficult. Ordered calomel and opium every three hours, and a blister to the abdomen.

Vespere.—She is evidently sinking; pulse 150; the extremities are warm, and the skin soft.

At one o'clock on the following morning she expired.

*Sectio cadaveris, thirty-three hours after death*. Head: There was slight congestion in the vessels of the brain, and, on cutting through its substance, a greater number of bloody points were observed than in a healthy brain. Thorax: The viscera appeared quite healthy. Abdomen: There was a pretty copious effusion of dirty-looking serum mixed with that curdy matter so common in these cases; the peritoneal vessels were in a few parts more turgid than natural, though by no means generally so. One of the round ligaments presented the appearance of inflammation. The external surface of the uterus was perfectly healthy; the internal was not examined, as I wished to have the uterus entire: it was of large size, and flabby; from its cavity, a quantity of coagula was expelled by pressure.

Case III. Mrs. S. was delivered on the 21st of February, after a very natural labour. I saw her on the 23d, and found that she had this day been seized with a rigor, followed by heat, pain in the belly, and profuse perspiration: her pulse was 120, but soft, and, since the occurrence of the perspiration, the pain had subsided. I was in hopes that this might turn out a mere case of ephemera, and, as there seemed no necessity for active measures, I prescribed small doses of calomel and opium, with a view of tranquillizing the system.

24th.—Much to my annoyance and astonishment, I found that the woman who acted as nurse had got her out of bed, and I found her sitting in a chair: she expressed herself easy.

25th.—Worse. Pulse 120, and with some slight degree of power; there is severe abdominal pain. Venæsectio ad ℥x. Capiat Cal. gr. ij.; Opii gr. i. tertia quaque horâ.

26th.—Head slightly affected by the opium; pain less; pulse 120; tongue furred. Hirudines xv. abdomini applicand., with constant fomentation.

The treatment of this case not being registered at the time, I cannot state what medicine was ordered, though I believe it was Mist. Salin. cum Ant. Tart.

27th.—Very much worse. Pulse 140; respiration difficult; there is a curious involuntary twitching of the muscles of the lip; tongue cleaner; abdominal pain very great, and there is much distention. This was relieved by an oil-of-turpentine draught; a large blister ordered to be applied to the abdomen.

In the evening, it was evident she was sinking, and therefore, although in great pain, I ordered her Ammon. Carb. gr. iij. omni horâ. About five o'clock the next morning, she expired in great agony.

*Sectio cadaveris, thirty-five hours after death.* The intestines were greatly distended with air; the peritoneum lining the cavity rather more injected than usual, that lining the bowels much more so; the convolutions were united by slight adhesions, by which also the omentum was bound to them; there was a large effusion of turbid serum, with a quantity of curdy matter. The uterus pale externally. The internal surface of the stomach appeared healthy: there was, however, a quantity of dark-coloured secretion contained within it. Effusion of the same character as in the abdomen had taken place in the chest, but it did not contain so much curdy matter. The lining membrane of the trachea was much injected.

Case IV. Mrs. K. was delivered of her third child on the 17th of January, after a very easy labour, and was doing very well till the 21st, when she was seized with a rigor, accompanied with pain in the uterus; pulse 140. Ordered twenty leeches to be applied to the abdomen. Calomel and opium every four hours.

22d.—She is quite free from pain; feels very weak and depressed; pulse 150. Was sitting up in bed, nursing her child. Bowels have been twice opened today.

In the night she was seized with vomiting, and, whilst her husband went to fetch a neighbour to her assistance, she expired. An inspection of the body could not be obtained, on account of the interference of the parish-officers.

Case V. Mrs. M. was delivered of her fifth child on the evening of the 24th of February. I saw her at half-past nine P.M. on the 26th, and found that she had had no bad symptoms till last night, when she was seized with violent pain in the abdomen, somewhat similar to afterpains, viz. occurring only at intervals, so far as regarded the violent paroxysms. In the night a rigor came on, followed by heat, and subsequently by perspiration. She is still complaining of great pain in the uterine region, both before and behind. The uterus is of large size. Pulse 124, and very feeble; the tongue had a brownish stripe down the middle, but was clean at its edges; pressure to a considerable extent could be borne without greatly increasing the pain, and she could turn in bed without much difficulty. The skin was dry, but not particularly hot; the lacteal and lochial discharges continue natural. She had experienced great uneasiness in the head, accompanied with dizziness and ringing in the ears. Capiat statim Hyd. Submur. gr. iij.; Pulv. Opii gr. ij. Utatur fotus abdomini.

Half-past one P.M.—Feels easier. Pulse 108; head tranquil; tongue more generally white. Has had no motion since delivery. Capiat statim Pulv. Rhei ℈i.; Potass. Sulph. ℈ij.; Aq. Menth. Piper. ʒxi., et post alvus respond. Pulv. Opii gr. iss.

Half-past four P.M.—Medicine not operated. Pulse 120, very powerless; pain relieved. Habeat statim enema commune cum Ol. Terebinth. ℥iss. To take the opium pill after the bowels have been moved. Tongue dry, and browner.

Half-past seven P.M.—No evacuation has taken place; has a feeling of tenesmus. Rep. Enema sine Terebinth. To take beef-tea.

February 27th, nine A.M.—Had two or three injections last evening, but no motion was procured till three o'clock this morning, which produced relief; after which she took the opium pill ordered yesterday. She is now in considerable pain at the bottom of the abdomen; pulse 120, but more firm; feels a disposition to sleep, but cannot; the lochial and lacteal discharges continue. Complains of thirst; tongue furred, but not dry; is much oppressed with wind. Capiat secundis horis Sodæ Tart ℈i.; Syr. Papav. ʒss.; Aquæ Menthæ Pip. ʒx.

Three P.M.—Pulse 140, probably somewhat accelerated by my sudden entrance, as she was at that period dozing; tongue brownish, and rather dry; the abdomen evidently distended with air, for, upon gently percuting it, a sound was emitted somewhat like a drum; the skin warm and dry, but not hot; countenance anxious, but not distressed; milk and lochia continue. Seeing the want of success which had attended the previous cases, in this instance I avoided very active measures, and endeavoured to temporise, in order to give nature a chance of doing what art did not seem able to effect. The chamomile bag previously ordered was directed to be continued; its exterior to be sprinkled with Ol. Terebinth.

Nine P.M.—She is no worse: pulse 120, and more steady; tongue brownish and dry. Has had a motion, and passed her urine; the milk and lochia continue. The chamomile bag with the turpentine was applied very hot, so as to produce considerable pain, and it had been therefore removed, for, on examining the abdomen, it was considerably reddened as if scalded, which was in fact actually the case. The breathing was rather hurried and laboured; skin warm and dry; there was great thirst. Ordered her a little beef-tea, and to take at bed time Cal. gr. v., Pulv. Opii gr. iij., with the hope of inducing sleep.

February 28th, half-past nine A.M.—Has had but little sleep, though she has been dozing; can turn much easier, but there is still pain in the belly, which is distended with air; there is also evidently an effusion of fluid into the abdomen. Pulse 128, and steady; tongue not quite so brown or dry; the lacteal and lochial secretions continue, though in small quantities. Wishes to have some boiled milk, which was allowed her. Breathing slower, more deep, and not so laboured. Rep. Sodæ Tart. ʒi. secundis horis.

Two P.M.—Not quite so well: pulse 136; tongue much more clear at the edges, the crust peeling off; secretions continue, but in sparing quantities; feels thirsty and qualmish at the stomach. Has had one motion; a considerable quantity of flatus passed with it. Capiat Haust. Effervescen. secunda vel tertia quaque horâ.

March 1st, half-past nine A.M.—Has passed a very bad night. Vomited frequently, the matter ejected being green and very bitter to the taste; has also had two or three loose offensive motions; abdominal swelling has increased, and there is much more tenderness on pressure; the pulse, however, has fallen to 118, and is more full. The countenance is very bad. She has not been able to take any food, in consequence of her irritable stomach. The tongue much the same as last night, viz. very red round the edges, with a thick white streak in the middle, but it is quite moist. The obvious indication was to relieve, if possible, the stomach, for which purpose I ordered her Hyd. Subm., Pulv. Opii āā gr. i. omni hora per quatuor vices; and a large blister to be applied to the epigastric region.

Half-past six P.M.—Sickness perfectly relieved; tongue nearly clean; pulse 130; very little pain; bowels also settled. Has had a good deal of uneasy dozing, perhaps the effect of the opium. Took a little tea, and a small piece of toast, and says she relished it. Ordered her to take slight nourishment, and to omit medicine for the night, there appearing no indication to be fulfilled.

March 2d, half-nine A.M.—Has passed an uncomfortable night, though she has had frequent dozings. Countenance yesterday and today exceedingly pinched, presenting much the appearance of a patient labouring under an attack of common fever. Appetite bad; mouth clammy; tongue dry, and of a brownish-red appearance; feels very weak. Has had a copious liquid evacuation, which is rather lighter than usual, but not particularly offensive. Blister has acted well. Abdomen very much distended with air. Has just taken a small quantity of boiled milk, but did not enjoy it. Head feels uneasy; pulse 128, and feeble. I determined to try the effect of the sulphate of quinine, of which I ordered her two grains, with five grains of capsicum, every hour, for four hours.

Seven P.M.—Evidently worse: pulse 130, and very feeble; tongue moist, but red; stomach and œsophagus feel very hot, which she attributes to the pills; great thirst, and a very distressing degree of restlessness. Capiat secunda quaque horâ Pulv. Opii gr. ij., Hydr. cum Cretâ gr. v.

She expired about eleven o'clock the next day.

*Sectio cadaveris, twenty-two hours after death.* When the cavity of the abdomen was opened, the intestines were observed to be considerably inflated, their peritoneal coverings much injected, and presented a dull lurid appearance; that portion of the membrane which lined the muscles of the abdomen was greatly injected in those parts which were in contact with the convolutions of the intestines; there was also a fulness in the vessels of the peritoneum generally. The surfaces of the peritoneum covering the intestines were slightly agglutinated by an unorganized substance. There was a large quantity of brownish, muddy effusion, mixed with a number of flakes, of a curdy consistence. The omentum greatly injected. The uterus pale and flabby externally, the follicles around its neck enlarged, and filled with a tough secretion; its substance pale and healthy, both without and within. On examining the peritoneum which lined the diaphragm, it was seen to be exceedingly injected, more so than at the lower parts: it was very easily peeled off the liver, which was soft in texture, as was also the spleen. There was a marbled appearance in the interior of the stomach, with numerous patches of extravasated blood under the mucous membrane. The mucous membrane of the rectum was partially inflamed. There was also slight unorganised adhesions between the two surfaces of the pleura.

CASE VI. Mrs. O. was delivered of her first child on the 26th of February, and was doing well till the 2d of March, when she experienced a rigor, followed by heat and intense pain in the abdomen: the pulse was rapid, though feeble. My pupil, Mr. WILSON, ordered her ten leeches to the abdomen, and calomel with opium through the night.

On the following morning, at nine o'clock, I saw her: the pulse was 140, and rather of a doubtful character, not altogether without power; the tongue was moist, the head very painful, and she vomited frequently. The abdominal pain has been relieved since the leeches were applied. Venesectio ad ℥x. To apply the chamomile bag wetted with oil of turpentine to the belly; and, with a view of relieving the sickness, to take small doses of Syr. Papav. with Soda Tart. every two hours.

Half-past two P.M.—Found her on her side in a sound sleep; pulse had fallen to 130, and very feeble. She appeared quite exhausted. Stomach irritable, but has retained her medicine.—Repetantur.

Nine P.M.—Pulse 136, and extremely feeble; tongue white, but moist; head heavy, scalp hot; can bear considerable pressure without flinching, but lies with her knees bent towards the pelvis. Lochial discharge has ceased; has had no motion; there is nausea, but no vomiting. She does not notice the child. The blood is slightly buffed, and cupped; the layer of buff only slightly adherent to the crassamentum.

March 4th, ten A.M.—Feels easier: has had four motions; pulse 110; tongue coated in the middle, but clean at the edges; feels low and faint. Plenty of lacteal, but no lochial discharge. Abdomen tender, but she can lie with her legs extended; head easy; nausea relieved. Has had a tolerable night.—Rep. Soda Tart.

Nine P.M.—Pulse 110. Expresses herself better. Tongue white, but more moist; there is no pain in the head; scalp rather hot. Complains of tenderness and soreness in the uterine region. Has had no motion.—Continue the chamomile bag and the medicine.

March 5th, nine A.M.—Not quite so well. Pulse 110, feeble; tongue white and moist; pain and uneasiness on pressure chiefly in the right groin; feels full and uneasy in the abdomen. She had taken, an hour ago, Ol. Ricini ʒij., which had not operated. I ordered it now to be repeated, conjointly with Ol. Terebinth. ʒv.

Ten P.M.—Pulse 104, rather fuller and regular: tongue white and moist. Has had three watery motions, of a brown colour; has felt several times during the day an inclination to vomit. There is a little increase of pain in the right side of the abdomen and groin; scalp cooler; plenty of milk.—Continue the chamomile bag and turpentine. Cap. statim Liq. Opii Sedativ. ♏xv., et repet. in nocte si opus sit. (Both doses were taken.)

6th.—Has passed a very comfortable day, though she slept but little during the night. Pulse down to ninety-eight, soft and round. Still pain in the abdomen on pressure; no sickness; plenty of milk, and has tonight a little lochial discharge. Has had no motion.—Capiat cras mane Pulv. Rhei ʒss.; Pot. Sulph. ʒi.

She has been asleep nearly the whole of the afternoon.

From this time she gradually recovered.

CASE VII. Was called to Mrs. K. at three o'clock P.M. on March 3d, who was delivered of her first child on the 1st instant. She had experienced several rigors, followed by intense heat over the whole surface of the body; great thirst; pain in the head; heat of scalp; flushed yet cheerful countenance; pulse 130, and rather feeble; considerable pain in the lower part of the abdomen; scarcely any lochial discharge. She has vomited frequently. As she was a young woman who had been previously healthy, I thought it safer to abstract about eight ounces of blood, which made her very faint. Her bowels had been freely opened yesterday. Ordered her Liq. Ammon. Acet. ℥ij.; Liq. Ant. Tart. ʒiij,; Syrup. Croci ʒiss.; Mist. Camph. ℥vi. M. cap. coch. ij. quartis horis.

Eight P.M.—Much relieved. Head easier; tongue white and furred; pulse 130, and feeble; great heat of scalp; abdominal pain less.—Pergat.

March 4th, twelve P.M.—Tongue much cleaner, and moist; less thirst; head easier. There is still considerable pain in the uterine region when pressed upon. No lochial secretion, and less lacteal, than yesterday. Was very restless during the fore part of the night. Diarrhœa then came on, which relieved her greatly. She can now lie with her legs extended without pain. Has much less thirst and heat; head perfectly relieved; pulse 114.—Rep. Mist. Salina.

Nine P.M.—Much worse. Soon after I left in the morning, the pain in the abdomen returned, and is now very great; the slightest pressure gives pain. Has had many liquid evacuations. The head is easy; pulse 128, and very feeble; tongue covered with a white fur; countenance anxious and flushed. She also complains of pain in the chest.—Applic. Empl. Lyttæ amplum scrobiculo cordis. ℞. Confect. Opiat. ʒiss.; Conf. Aromat. ʒij.; Mist. Cretæ ℥viij. M. capiat coch. ij. omni horâ.

March 5th, seven A.M.—Excessive abdominal pain always present, but aggravated at intervals; pulse 120, and feeble; tongue brown and dry. Describes her pains as shooting from one side to the other, and says they are much worse than labour pains. Headach gone, but scalp still hot; has vomited once; when any fluid is taken, she feels as if she was bursting; her countenance was anxious, and she was constantly moaning and crying out. Diarrhœa is checked; lacteal secretion continues. Ordered the chamomile bag, with turpentine sprinkled over it, to be applied to the abdomen, and to take Liq. Opii Sedat. ♏xv., Aq. Cinnamomi ℥i. statim. This was not taken till half-past eight.

Half-past nine A.M.—Much the same. Rep. Haust. Anodyn., and to take another with twenty-five minims of the Liq. Sedat. in an hour from this time, if the pain continue.

Two P.M.—Has taken both doses of the Liq. Opii Sedat. and feels much easier: the pulse has fallen to 104, and she is free from pain, except when pressure is applied. Can turn with less difficulty.

Half-past nine P.M.—Not quite so well. Pulse 120; tongue rather more moist, but still brown; has had no motion; feels pain on pressure. Has vomited several times. Secretion of milk increased.—Capiat hora somni Syr. Pap. ʒi.; Liq. Opii Sedativ. ♏xx., et repet. in nocte si opus sit. Capiat mane primo Olei Terebinthinæ ℥ss., Olei Ricini ʒiij.

6th, nine A.M.—Took both doses of the opium, but had no sleep: this she attributes to her being disturbed by her child. Vomited soon after taking the turpentine. Pulse 120; tongue streaked, white and brown; abdominal pain seems extending higher up, being now worse in the hypochondrium. Has had no motion.—Habeat statim Enema commune, et capiat omni horâ vel horæ dimid. Antim. Tart. gr. $\frac{1}{4}$.

Half-past one P.M.—Worse. Pulse 126; tongue brown and dry. The injection brought scarcely any thing away with it. There is great pain in the hypochondriac regions, the inflammation apparently extending to the peritoneal surface of the diaphragm, as there is great pain on respiration. Scarcely any milk.—Rep. enema, and apply warm turpentine cloths to the parts in pain.

In the evening the pain had increased, and the pulse was rising. She continued in great torture till near midnight of the 7th, when she expired. For twenty-four hours before her death, relief was attempted, though in vain, by the exhibition of large doses of Battley's solution of opium.

*Sectio cadaveris, sixteen hours after death.* The external appearance of the body was much changed; the countenance pinched, the lips livid; the hands clenched and blue. Different parts of the body also presented a mottled bluish appearance, similar to what is frequently observed in children who have died in convulsions. The muscles, when cut through, appeared of a dark colour, and the intestines greatly distended with air. The peritoneal covering of the intestines was very turgid with blood, but that lining the abdominal muscles not so much. There was a large effusion of the same turbid serum which was observed in the other cases, mixed with large flaky deposits: none of these were situated between the folds of the intestines; they were perfectly free, having no kind of adhesion between them. The ovaries were exceedingly soft and pulpy, and, when pressed between the fingers, seemed to be resolved into the same kind of curdy matter which was effused into the abdomen. The liver was immensely enlarged, but healthy.

As the friends were anxious that the body should be disfigured as little as possible, the ribs were not divided, but an opening was made through the diaphragm into the chest. The lungs appeared healthy, but large masses of curdy substance were adhering to the costal pleura, the same in appearance as in the abdomen.

Case VIII.—Mrs. R. was delivered of her seventh child on the 28th of February, after a very easy labour. Eight hours afterwards, she appeared low and desponding, and was covered with a profuse perspiration; the pulse soft and natural. She complained of pain in the side of the chest. The uterus hard and of large size; lochia natural; secretion of milk not commenced. There is heaviness and a little wandering in the head, probably caused by a dose of opium given her after she was placed in bed. She has a very irritating, hacking sort of cough, which lasted almost incessantly for several hours after delivery.—Applic. Emp. Lyttæ lateri dolent, et cap. Mist. Salin. quartis horis.

March 1st.—About twelve o'clock had a rigor for about twenty minutes, followed by heat and perspiration. Has had a gnawing pain in the uterus all night; felt it before the rigor came on; is increased by firm pressure. The uneasiness in the head still continues; pulse 120, regular and round; tongue moist, with a slightly brownish fur; has frequent flushes of heat, and sickness at the stomach, with retching; experiences pain when pressure is made on it; perspires much. Fluid can be felt in the lower part of the abdomen. Chest much relieved by the blister.—Apply chamomiles to the abdomen.

Four o'clock P.M.—Pulse 140; tongue moist and white. Has had a good deal of purging, the stools black and offensive; vomited about two pints of a viscid yellow fluid. Head giddy and wandering. Has taken nothing but gruel and barley-water.—℞. Haust. effervescens p. r. n. sumend. Hirudines viij. temporibus.

2d, eleven A.M.—Has had no sleep all night, and has been slightly delirious; complains of pains in the limbs and head. The leeches were not applied till this morning, and then only four of them. Pulse 132; tongue rather coated, but not dry; no lochia. Vomited up a brown yellowish fluid; but, after taking one of the effervescing draughts, the secretion became green.—℞. Hyd. Submur. gr. iij.; Ext. Coloc. c. gr. iv. fiat pil. h. s. s. ℞. Æth Sulph. ℥iss.; Aq. ℥ivss. M. fiat lotio capiti applicand. V.S. ad ℥xij. Emp. Lyttæ scrobic. cordis.

Four P.M.—Pulse 120; tongue moist and clean; head hot and heavy: no pain in the abdomen; no lochia nor milk.—℞. Sulph. Magnes. ʒiss. secundis horis.

Nine P.M.—The retching has continued, more or less, all day. The medicine has produced two black and offensive motions. She feels very weak and low. Ordered her to take some beef-tea. ℞. Liq. Opii Sedat. gtt. xx. h. s. s.

3d, ten A.M.—Pulse 120; cerebral symptoms relieved; tongue clean and moist; the sickness has returned; has had a little sleep.—℞. Sodæ Tart. ʒss.; Syr. Pap. Alb. ʒi.; Aq. Menth. Pip. ℥iss. M. fiat haust. omni horâ sumend.

One A.M.—Has a troublesome cough; sickness has continued till within the last hour; skin moist and natural. She feels much better, and has had no motion since last night.

Three P.M.—Pulse 130; tongue tolerably clean; feels sick; more pain and heat in the head.

Nine P.M.—Pulse 110, weak, and without power, increased by the slightest cause. Tongue white, coated, and moist. No pain in the abdomen, which is greatly distended. The uterus is very much enlarged. She has dyspnœa, with a mucous rattle; skin moist; very little milk. Bowels not opened today.—℞. Liq. Opii Sed. gtt. xx.; Spt. Ammon. Aromat. ʒss.; Aq. Menth. Pip. ʒx. M. fiat haust. horâ somni sum. Rep. Lotio capiti.

4th, twelve A.M.—Bowels have been relieved, the motions whitish. The draught procured but little sleep, and, when she awoke, she was teased with rattling in the throat; has pain in the stomach on pressure; dyspnœa still continues; pulse 130, small and vibrating; tongue white and coated. Head feels relieved, though the scalp is hot.—℞. Liq. Ammon. Acet. ℥ij.; Liq. Ant. Tart. ʒiij.; Syr. Croci ʒiss.; Aquæ Piment. ℥vi. M. capiat cochl. magn. ij. quartis horis.

Half-past nine P.M.—The pulse has risen to 160, undulating and powerless; tongue moist, white and coated; no abdominal pain. Has diarrhœa; the motions and urine pass involuntarily. Ordered her some arrowroot and port wine. ℞. Conf. Opiat. ʒiss.; Conf. Aromat. ʒi.; Mist. Cretæ ℥viij. M. capiat ℥i. secundis horis.

In the night she became convulsed, and showed great delirious excitement; and next morning, a little before eight o'clock, she expired.

*Sectio cadaveris, twenty-five hours after death.* The body was quite inodorous. The blood-vessels of the dura mater were slightly turgid, and there was a generally enlarged state of the vessels of the brain, with a very small quantity of fluid in the ventricles. There was some fluid in the pericardium. The lungs appeared healthy in structure, though injected with blood in some parts: they were much pressed out of their natural position by the enlarged state of the liver and spleen. The peritoneum was generally injected; there were large depositions of a flaky substance. The convolutions of intestines were connected together by a soft layer of gelatinous matter, easily broken through. The uterus was of large size, with a slight blush on its external surface: the substance, however, was firm and pale. The effusion of dark-coloured fluid very decided, though less in quantity than most of the other cases. The fallopian tubes were much injected. The spleen and liver were very much enlarged, but healthy in structure.

Case IX. Mrs. G. was delivered, on the 18th of March, of her second child. On the 19th, she did not seem very well, though there were no urgent symptoms: the pulse hurried; the lower part of the abdomen a little tender, though considerable pressure could be borne.

On the 20th, she was much the same. Has taken two doses of castor oil, without having had a motion; has had two or three creeping sensations, followed by flushings and perspiration. In the evening, the bowels were relieved, and she expressed herself very comfortable: pulse, however, not reduced. She is quite free from abdominal pain; secretion of milk copious.—Capiat statim Hydr. Submur. gr. iij., Pulv. Jalap. gr. xv.

21st, mane.—Has had several motions, and says she is better, though her pulse remains at 120; is thirsty; tongue rather white; lochia in small quantities, and fetid.—℞. Liq. Ammon. Acet. ℥ij.; Liq. Ant. Tart. ʒiij.; Syr. Croci ʒiss.; Mist. Camph. ℥vi. capiat coch. mag. ij. quartis horis.

Two p.m.—Feels much worse. Has had another creeping sensation, hardly amounting to a chill, followed by flushing and uneasiness in the head; the pulse 140, and rather feeble; the tongue much more white, but not very dry. There is now a diffused tenderness over the abdomen, and the intestines are evidently inflated with air: can turn, however, without much difficulty. I placed her in the erect posture, and removed about twenty-four ounces of blood, which made her exceedingly faint, and, in order to keep up this effect, I placed the pillows so as to prevent her head from again slipping down. I gave her, during the bleeding, Hydr. Submur. gr. iij. Pulv. Opii gr. ij., and directed a dose of the saline mixture previously prescribed to be given her in two hours. She felt most oppressively faint, her pulse sank to 120.

Five p.m.—Still remains faint. Pain in the uterine region gone; bears pressure better; head easier; pulse 136, and rather feeble; she feels sleepy.—Applic. abdomini Hirudines x. Capiat omni horâ Hyd. Submur. gr. i.; Pulv. Opii gr. ss.; Antim. Tart. gr. $\frac{1}{6}$, in pil.; et cras mane Ol. Terebinth. ʒij. cum Ol. Ricini ʒvi.

Nine p.m.—Leeches bled well. Feels perfectly free from pain; tongue white, but a little cleaning at the tip; pulse 140, and thready; thirsty, skin rather dry. Has had no sleep. Continue the calomel and opium.

22d, half-past seven a.m.—Had about four hours' sleep in the night. Has taken ten doses of the pills; took the draught at half-past five, but has had no motion. She was perfectly easy till half-past six, when the pain returned, which was chiefly situated in the right iliac region: can bear considerable pressure without producing violent pain, though there is a degree of tenderness; skin dry. Secretion of milk and lochia continue, though in sparing quantities. Tongue white; countenance, since the bleeding especially, almost blanched. Can turn in bed tolerably easy, and talk with a cheerful tone of voice. Pulse rather more than 140, very small and thready.—Habeat statim Enema commune cum Ol. Terebinth. ℥iss.; and, if not easier after its operation, to apply eight more leeches to the right side of the abdomen. Capiat omni horâ Hyd. Subm. gr. ij.; Pulv. Opii gr. ss.; Ant. Tart. gr. $\frac{1}{3}$, in pil.

Twelve M.—Worse: pulse 150. Has had two injections, yet nothing passed but air, at least no feculent matter. Tongue cleaner, countenance very anxious; abdomen tumid. Has taken four doses of the pills. To take half a pill every hour.

Half-past two.—Much the same: has passed some wind, but no feces. Gave her an enema, which returned immediately, with a small portion of feculant metter: feels as if she was about to have a motion. Abdomen is now soft and flaccid, and the tumefaction has subsided.

Half-past six.—Soon after I left, the patient had one loose motion, but she is still in pain; has also passed a large quantity of water. Pulse 160; tongue more moist; very thirsty; skin hot, but not quite so dry. I resolved to resume the calomel pill, and therefore ordered her Hydr. Submur. gr. ij.; Ant. Tart. gr. $\frac{1}{3}$; Pulv. Opii gr. ss. statim, et repet. horâ nonâ. She expresses a great desire for sleep; abdomen again distended.

She went on in much the same way till the next evening, when she died.

*Sectio cadaveris, thirteen hours after death.* The abdomen was much distended. The peritoneum covering the intestines highly injected, that lining the muscles but slightly. The intestines were enormously inflated, excepting the ascending portion of the colon, which was greatly constricted. The substance of the uterus was healthy, but its peritoneal covering had a slight blush on it. The convolutions of the intestines were quite free, there being no deposit between them. The usual effusion in the peritoneal cavity was seen in abundance. There were also patches of inflammation on the peritoneal surface of the liver.

CASE X. Mrs. L. was delivered at seven P.M. on the 25th of March, and was quite comfortable till three o'clock in the afternoon of the 27th, when she was seized with a rigor, followed by heat and throbbing in the head. One of my pupils, Mr. BUCHANAN, saw her at half-past six, and found the skin hot, face flushed, pulse 140, tongue clean and moist; considerable pain, chiefly referred to the back; is very thirsty. He removed about twelve ounces of blood. ℞. Ol. Ricini ℥i. statim sum.

When I saw her at eleven, the pulse was still 140, and feeble; the countenance pale; the uterus enlarged, and great pain is felt when pressure is made on it; the lacteal and lochial discharges natural; she complains of much thirst. The bleeding had caused a great degree of faintness, which remained for a considerable time, but is now in some degree subsided. As the attack was so recent, I untied the bandage, and allowed about eight ounces more blood to flow, when she again became very faint. I recommended her head to be kept high, with a view of keeping up syncope. She had one motion whilst we were there, which was rather offensive and very costive. Capiat omni horâ Hyd. Submur. gr. ij.; Pulv. Opii gr. i.; Ant. Tart. gr. $\frac{1}{8}$.

28th, eight A.M.—Countenance quite blanched, lips pale; remained very faint after the bleeding. Has dozed a good deal. Tongue natural, abdomen easier, and not tympanitic: she flinches, however, when pressure is applied to the uterus. Pulse 130, and rather vibrating, giving to the finger an hemorrhagic twang. Had another large costive motion last night. Ordered the Cal. and Ant. Tart. to be continued without the opium, as the bowels were confined, and she seemed a little stupified by it.

Three P.M.—Much the same: pulse 140, tongue moist. Has had three motions: feels very faint. Not much abdominal tenderness, except when pressed upon.—Rep. Pil. cum Opio statim, et sine Opio omni horâ, if the bowels be quieted.

Eight P.M.—Seems worse. Pulse 140; rather more abdominal tenderness; is very restless; the lochial and lacteal discharges continue; is thirsty. Has had no motion since three o'clock. Her head seems a little wandering: says she is in no pain, but does not know why she is so restless. —Capiat secundis horis Cal. gr. ij., Pulv. Opii gr. $\frac{2}{3}$. Infricand. Ung. Hydr. fort. inter femora quartis horis.

29th, seven A.M.—Exceedingly faint, and has great pain; pulse 140, tongue rather brown and more dry. When asked how she felt, she replied rather sharply, "quite well."

Cannot bear pressure over the abdomen; is thirsty; has taken gruel through the day; feet cold; urine and feces pass involuntarily. She has taken the calomel pills through the day, and, as her bowels were relaxed, she was ordered a mixture composed of Conf. Aromat., Conf. Opii, et Aq. Menth. Piper. The ointment has not been rubbed in to the extent I wished: mouth not at all affected.

Ten P.M.—There is an evident amendment: pulse 120, skin soft and warm. Has had two drachms of Ung. Hydr. fort. rubbed in; tongue rather brownish, mouth not affected. Is in but little pain, except when pressed upon, and then she flinches greatly; feels very weak and faint. Wishing to give the mercurial plan a trial, and fearing to disturb her bowels by the internal administration of calomel, I ordered the fumigation of Hydr. Sulph. rub. ʒi., and directed it to be repeated twice in the night.

30th, seven A.M.—Pulse 130; has passed a very restless night; urine and feces pass involuntarily; eyes sunk. Used the fumigation once during the night, but refused it a second time. Is thirsty, and has continued moaning for the last forty-eight hours.

Three P.M.—Found her evidently dying: extremities cold, pulse hardly perceptible. In the evening I received a message, saying she was dead. An examination could not be obtained.

Case XI. Mrs. W. was delivered March 26th, in the morning: she was doing well till ten o'clock on the following morning, when she was seized with a rigor, followed by heat and intense sore pain in the abdomen. One of my pupils called at three o'clock, and immediately removed about ten ounces of blood, which he states softened the pulse, but did not diminish its frequency, and, when he left her, it beat 140 strokes in the minute.

Five P.M.—The pulse is still the same, feeble and undulating; tongue clean and moist, the skin natural; there is great abdominal pain in the situation of the uterus and round ligaments, but in other parts pressure can be borne; the uterus is also enlarged; lochia lessened in quantity; abdomen distended, but says she was so after her last child. As the disease at present is confined to a comparatively small extent of the abdomen, and as the attack was recent, I considered it justifiable, notwithstanding the feebleness of the pulse, to repeat the bleeding, which I did to about eight or ten ounces: she then felt faint. I desired her to be kept in a semi-erect posture, to have twenty-four leeches to the abdomen, and to take every hour Hydr. Submur. gr. ij.; Pulv. Opii gr. i.; Ant. Tart. gr. $\frac{1}{5}$.

Ten P.M.—There had been some delay in the application of the remedies: the leeches had just come off, and she had only taken two doses of the pills: was lying upon her side, and says she was easier than when upon her back; pulse 124 and soft, tongue clean and moist. Neither of the portions of blood was either cupped or buffed. Rep. remedia.

March 28th, seven A.M.—Has taken ten doses of the pills, and has dozed considerably during the night. Uterus diminished in size, and is less tender; there is pain in the course of the descending colon; abdomen tympanitic; pulse 120 and soft; has lost the undulating feel. Leeches have bled profusely. Has had one motion in the night, which was natural: head easy. No milk, but says she seldom has any till the third day.—℞. Hydr. Submur. gr. iss.; Pulv. Opii gr. ss.; Ant. Tart. gr. $\frac{1}{6}$, fiat pil. omni horâ sumend. Applicand. abdomini cataplasma anthemid. cum Ol. Tereb. Lochia continue.

Two P.M.—Says she is easier. I found her sitting upright in bed, taking some gruel: can bear pressure better; pulse 124, tongue tolerably clean and moist, abdomen tympanitic.

Ten P.M.—Found her much worse: head confused; was constantly moaning; abdomen much more distended, pulse 130. Ordered her Hirudines viij. abdomini, and the pills with gr. $\frac{1}{4}$ of opium, instead of gr. ss. Ung. Hydrar. fort. ʒss. to be rubbed in between the thighs.

29th, noon.—Much surprised at finding her alive: her pulse upwards of 140, and so feeble as to be felt with great difficulty; abdomen tender, hands and feet getting cold.—℞. Ammon. Carb. gr. xxiv.; Tinct. Card. co. ʒiij.; Mist. Camph. ℥vi. M. capiat coch. ij. omni horâ. Bottles of hot water to her feet. In about two hours after this, she expired. No examination of the body was allowed.

CASE XII. Mrs. H. was delivered on the 28th of March, in the morning, after a remarkably easy labour. When she was visited at two o'clock on March 30th, she was found complaining of great pain in the abdomen, pulse ninety-two, and rather powerful. Sixteen ounces of blood were removed. ℞. Hyd. Subm. gr. ij., Pulv. Opii gr. ss., Ant. Tart. gr. $\frac{1}{6}$, M. fiat pil. omni horâ sumend. Infricand. Ung. Hyd. fort.

Seven P.M.—At this time the symptoms are rather increased: pulse 108 and soft, great pain on pressure on the uterine surface, low down behind the symphysis pubis; tongue clean and moist. The ointment has been rubbed in, by mistake, on the lower part of the abdomen, instead of the thighs. Soon after she was bled she had a very distinct rigor, followed by great uneasiness in the head. Has once vomited. Applicand. Hirudines xx. abdomini.

In the evening her pulse had risen to 136. The calomel and opium to be continued without the Ant. Tart. Mercurial fumigation was used at eleven o'clock, (Hyd. Sulph. rub. ʒi.) and ordered to be repeated every four hours.

31st, seven A.M.—The report at this time is, that she is easier: pulse 124. Has had no motion since the 29th. The mouth is not affected by the mercury, (has taken, since yesterday afternoon, fifteen two-grain doses of calomel, besides three mercurial frictions and three fumigations.) The fumigations were ordered to be continued. ℞. Hyd. Subm. gr. iij., Pulv. Opii gr. ss. fiat pil. omni hora sum. ℞. Ol. Ricini ℥ss. statim sum.

One P.M.—The pain in the abdomen is nearly gone: she can bear considerable pressure without flinching; tongue clean, pulse 130. Has slept for a considerable time, and had one loose motion, which suddenly passed away in the bed. A little sponginess of the gums is perceptible, though she says her mouth is not sore. Lochial discharge natural, but no milk. Has fumigated once since seven o'clock, and taken two doses of the three-grain pills. Ordered the fumigation to be repeated at two o'clock, and to take a pill every three hours till the mouth is affected.

Nine P.M.—Has had a good deal of refreshing sleep: pulse 138, full and soft; says she is in no pain, except when pressure is made on the umbilical region. The uterine surface, which was at first so tender, is now in its natural state. Has fumigated once, and taken two pills (three grains of calomel in each,) since the last report: her mouth feels rather sore, and the gums appear a little inflamed. Has had another loose motion.—Capiat tertiis horis Cal. gr. ij., Pulv. Opii gr. i.

April 1st, mane.—Worse: pulse 140, and has a great increase of pain. Her mouth is sore, and therefore two pills only were given during the night. Has had two motions. —Rep. Pil. et applic. Hirud. xx.

One P.M.—Evidently worse: pulse 140 and more feeble; the pain great; the countenance parched, and exhibiting great distress; the eyes sunk, tongue brown and dry; the mouth affected by the mercury; the bowels purged.—℞. Conf. Opiat. ʒiss.; Conf. Aromat. ʒij.; Aquæ Menth. Pip. ℥viij. M. capiat coch. ij. omni horâ.

The medicine relieved the pain and the relaxation of the bowels, but she died on the following morning.

*Sectio cadaveris, seven hours after death.* The abdomen was remarkably prominent, owing to the viscera being greatly distended with air; both peritoneal surfaces, viz. that lining the muscles and that covering the bowels, were greatly injected; the gallbladder immensely distended by an accumulation of bile; the omentum was turned up, its inferior surface resting upon the gallbladder. No adhesions between the convolutions of the intestine, and, although the effusion of serum into the abdominal cavity was large, there was but little curdy matter mixed with it. The veins of the uterus were pale, showing no marks of inflammation; its substance pale, and both surfaces healthy. The stomach contained a large quantity of black fluid, and on the mucous membrane there was a number of vascular patches: the same appearance was also observed in the duodenum.

Case XIII. Was requested, on March 29th, about three o'clock in the afternoon, by one of my pupils (Mr. Clutterbuck), to visit Mrs. P., who was in labour, and who seemed to be in a very low state: she was nearly as white as the sheet which covered her; the pulse about 100, and extremely feeble; the skin relaxed and cool, the vagina also relaxed and nearly cold. There was a constant pain in the abdomen, but no effect was produced by it on the os uteri, which was not half dilated; the remaining portion felt hard and thick. There was a constant drain of blood from the uterus, but not amounting to hemorrhage, though, in her exhausted state, it was more than she could spare. The membranes containing the liq. amnii were unbroken. She had been in a fainting condition several times: the discharge had not lasted more than an hour and a half. I gave her Tr. Opii ʒi. with a view of relieving the pain, and directed her to have a small quantity of nourishment frequently given her.

In the evening she felt better; was more warm; the pulse 108, and scarcely perceptible. Capiat Ext. Hyoscy. gr. x. horâ somni, et repet. post horas iv.

30th, half-past five a.m.—Was sent for in great haste, and before I arrived at the house the child was born, but dead: there was no hemorrhage, and she expressed herself tolerably comfortable. I saw her again at half-past nine: she was in the same state, but cheerful; she complained of the pain she had before and during labour, which was situated at the lower parts of the abdomen. She took through the day an anodyne mixture.

31st, eleven A.M.—Very ill: pulse 140; tongue whitish and rather dry; bowels very much distended; had a chill in the night; complains of pain in the abdomen, though considerable pressure can be borne. I considered the disease in such a constitution to be a mortal one, and yet I wished to try something: I gave her Cal. gr. x., Pulv. Opii gr. ij. and ordered Ol. Ricini ℥ss. to be given in four hours. Fotus communis abdomini.

Six P.M.—Remains in the same state: pulse 140, and feeble. Has had no motion. Capiat statim Ol. Ricini ʒiij., Ol. Terebinth. ℥i. Applicand. abdomini Emp. Lyttæ, et illin quartis horis inter femora Ung. Hydr. fort. ʒiij. Has had scarcely any lochial discharge since her delivery.

Ten P.M.—Pulse 140, small and wiry; complains of great pain, though a certain degree of pressure can be borne; tongue rather whitish, but not dry: has had no motion. She vomited up the greater part of the turpentine draught. Habeat statim Enema commune cum Sodæ Muriatis coch. magno. Rep. Ung. Hydr. et capiat Cal. gr. ij., Pulv. Opii gr. i. secundis horis.

She lingered till two o'clock in the morning, when she died. No examination was allowed.

One case of puerperal convulsions has occurred, which happened to a young woman in labour with her first child. When I arrived at the house, about eleven o'clock, I found her labouring under a most violent attack of convulsions, which had been repeated, with little or no intermission, for about an hour. The pupil in attendance (Mr. Morris) had removed about twelve ounces of blood, without the slightest relief, and had several times dashed cold water in her face without effect. The pulse, though rapid, was feeble; the lips had a leaden hue, and there were no marks of determination of blood to the head, which made us hesitate to repeat the bleeding. As, however, the force of the convulsions seemed increasing, another vein was opened, and twelve ounces more blood abstracted. No good effect, however, being observed to follow this practice, and as the parts of generation were lax and dilatable, it was agreed, with the concurrence of my friend and colleague, Mr. Doubleday, to finish the delivery by turning. My own opinion was rather in favor of craniotomy, as the female was of small size, and it was her first child; but, it being thought right to give the child a chance of life, I yielded, and agreed to turn. This operation was effected with some difficulty, for the head did not readily recede, but came down with the feet: at length I succeeded, and brought the child through the external parts. The placenta was soon expelled.

After delivery, the convulsions continued, and I left her about twelve o'clock to the care of Mr. Morris, who reported that soon afterwards a more violent convulsion occurred than before, accompanied with heat of scalp and flushing of the face, and that he, in consequence, removed about eight ounces more blood; and this last bleeding was followed by a great improvement in the symptoms. I saw her soon afterwards, and finding her head painful, and the scalp still hot, I ordered her eight leeches to the temples; the head to be kept cool by an evaporating lotion; and a Liq. Ammon. Acet. mixture.

From this time there was no convulsion, and she recovered without any untoward symptom; her chief sufferings arising from her tongue, which had been injured by her teeth during the paroxysms.

One case of active inflammation of the peritoneum has occurred, which was not very easily subdued: repeated bleedings, with the administration of calomel, so as to affect the mouth, at length proved successful. This female's pulse never rose above 100, and was attended with a very considerable degree of power.

In two or three instances, hemorrhage to a certain extent supervened, but in no case was it attended with any dangerous symptoms.

Several cases of what (for want of a better term) I have been in the habit of calling irritable peritoneum have occurred; the affection consisting of a very painful state of the membrane, the patient flinching on the slightest touch: there is, however, in general, no very great hurry of the circulation; the tongue is nearly clean and moist, and there is no great difficulty in turning round in the bed; the skin is also soft and a little damp, and the countenance does not exhibit that anxiety which it does in puerperal inflammation. Long continued fomentations, with calomel and opium, have invariably effected a cure.

Upon reviewing the cases of puerperal fever, some observations naturally arise, but the length of the present paper precludes me from making them at present: they must therefore be deferred for a succeeding Number of this Journal.

93, *Bartholomew Close; July* 1830.

